# Value of Neuroradiology Second Reads of CT Scans for Hyperparathyroidism

**DOI:** 10.3390/jcm14092865

**Published:** 2025-04-22

**Authors:** Javier Bravo Quintana, Michael Bouvet, Jennifer Chang, Julie Bykowski

**Affiliations:** 1 Department of Surgery, UC San Diego Health, San Diego, CA 92103, USA; jbquintana@health.ucsd.edu (J.B.Q.); mbouvet@health.ucsd.edu (M.B.); 2 Department of Radiology, UC San Diego Health, San Diego, CA 92103, USA; jenchang@health.ucsd.edu

**Keywords:** parathyroid adenoma, primary hyperparathyroidism, second opinion, second read, parathyroidectomy, minimally invasive surgery

## Abstract

**Background/Objectives**: Parathyroidectomy is a curative procedure for primary hyperparathyroidism, and multi-phase CT is an integral part of surgical planning. While patients may be referred to centers specializing in endocrine surgery, their imaging may be performed at other facilities without the same high-volume expertise. **Methods**: A retrospective review was performed of radiologist second reads of outside neck CT imaging in patients with hyperparathyroidism referred for surgical management. **Results**: The initial outside report was 59% sensitive for localization of parathyroid adenoma in the 74 patients with surgical pathologic confirmation. Second reads of the same CT scans correctly identified the parathyroid adenoma in an additional 24% of patients, for a total sensitivity of 83%. For the 23% of patients with pathologically confirmed multi-gland involvement, the initial outside report was 21% sensitive for lesion detection, and the second read of the same scans was 68% sensitive. **Conclusions**: Endocrine surgeons should be aware that community-based radiology interpretation of neck CT may be less sensitive than reported series from academic and high-volume practices. In the present study, interpretation via second read of outside CT scans by a neuroradiologist engaged with the endocrine surgery service line increased the sensitivity of detecting candidate lesions, both for single-gland and multi-gland involvement. While it is preferred to have preoperative imaging and interpretation within the same high-volume center as the surgeon for consistency of imaging quality, experience and communication, radiologist second reads deserve financial and service line support when that is not possible given the impact on surgical planning and patient care.

## 1. Introduction 

Primary hyperparathyroidism results from excess production of parathyroid hormone by abnormal parathyroid glands. This in turn elevates serum calcium levels, which may increase fracture risk due to bone resorption and contribute to gastrointestinal symptoms (nausea, constipation), nephrolithiasis and neuromuscular symptoms. While many patients remain asymptomatic despite high serum calcium levels, surgical intervention provides the only curative treatment for primary hyperparathyroidism and is indicated for symptomatic patients.

The American Association of Endocrine Surgeons recommends that imaging results should not be used as criteria for diagnosis and surgical referral but rather as part of operative planning to identify anatomic position [1]. Multi-phase neck computed tomography (CT) including non-contrast, arterial contrast phase and delayed “washout” phase has allowed for more precise localization of parathyroid adenoma for traditional and minimally invasive surgical planning in patients with primary hyperparathyroidism. Larger series with pathologic correlation have reported >90% sensitivity for lateralizing single-gland involvement and >80% sensitivity for correct quadrant localization [2,3,4]. Smaller series of patients with failed surgery or unsuccessful localization with ultrasound or nuclear medicine studies have also reported multi-phase parathyroid CT lateralization sensitivities of 75–84% [5,6,7].

While multi-phase CT is intended for operative planning rather than diagnosis of parathyroid adenoma [8], many patients undergo neck imaging prior to surgical referral. Additionally, patients may have imaging studies performed at outpatient imaging centers rather than at the same medical center as the treating surgeon due to referral patterns and insurance coverage. The use of multi-phase parathyroid neck CT protocols has increased over the past 20 years [9,10]; however, it is still not a routine order at all community-based imaging sites. Separately, the reported sensitivity of multi-phase CT for adenoma localization is based on a cohort of experienced neuroradiologists at high-volume centers for parathyroidectomy.

As an academic multidisciplinary medical center with high-volume referral for parathyroid surgery, we provide second-read review of outside CT scans rather than automatically requiring re-imaging prior to surgery. This study evaluated the impact of the neuroradiologists’ second reads for surgical planning prior to parathyroidectomy for hyperparathyroidism.

## 2. Materials and Methods

A retrospective review of the radiology reporting database from November 2019 to July 2024 was performed, identifying all second-read requests for review of initial outside-facility neck CT. Exams requested for evaluation of primary hyperparathyroidism were identified. Second-read reports created by the UCSD neuroradiology faculty at the time of clinical care were compared with the original outside radiology reports archived in the electronic medical record when available. Patient demographic data and operative and pathology results were obtained from the electronic medical record and compared with the initial outside and second-read interpretations for concordance. Imaging findings were reviewed by two neuroradiology faculty, each with >10 years of experience interpreting parathyroid neck CT scans (JB, JC). Outside facility radiologists were cross-referenced with the American Board of Radiology website to verify certification status (https://www.theabr.org/myabr/find-a-radiologist, accessed on 15 April 2025). The chi-squared test was performed for comparisons.

## 3. Results

### 3.1. Referral and Second-Read Utilization

A total of 97 second-read reviews were performed of outside-facility preoperative neck CT scans for patients with hyperparathyroidism, compared to 490 parathyroid neck CT scans performed in house during the same time. Original outside imaging was performed across 29 different locations. A multi-phase parathyroid protocol was performed in 66 (68%) of the outside CT studies. Image reconstruction ranged from 0.6 to 3 mm slice thickness in axial, coronal and sagittal planes. Only 16 studies (16%) included the <1 mm source images which could be reconstructed at the PACS workstation.

Original interpretations were performed by 37 board-certified radiologists; the radiologist was not identified on three of the available reports. Sixteen (43%) were confirmed to have an additional neuroradiology certificate of added qualification (CAQ). Second reads were performed by three board-certified radiologists with a neuroradiology CAQ, two with >10 years of experience and one with >5 years of experience interpreting parathyroid neck CT scans.

### 3.2. Patient Demographics and Presentation

Operative reports and pathology results were available for 74 patients. Demographic information is included in Table 1. Most patients were female (79%) and over 65 years of age (66%), with a mean age of 68.0 years. A total of 93 hypercellular parathyroid adenomas were confirmed in 72 patients. One patient had parathyroid carcinoma, and one patient had normal parathyroid glands on final pathology. 

Serum parathyroid hormone (PTH) levels and serum calcium were measured preoperatively and postoperatively for surveillance. The average preoperative serum PTH was 123.3 compared to 31.6 postoperatively; the mean change in serum PTH was −91.66 (*p* ≤ 0.05). Average preoperative serum calcium levels were 10.5 compared to 9.4 postoperatively; the mean change in serum calcium was −1.08 (*p* ≤ 0.05).

### 3.3. Sensitivity of Initial and Second Read 

**Figure 1:**
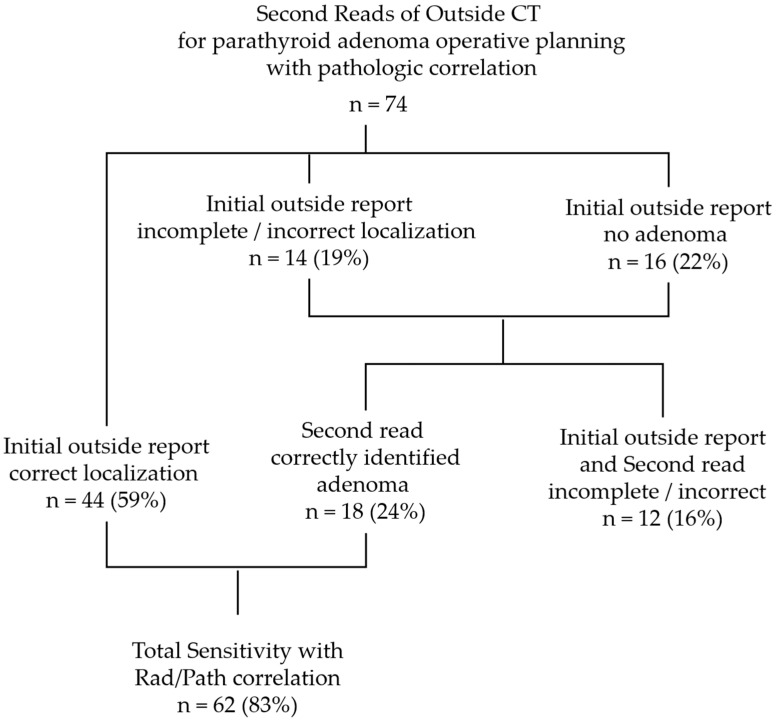
Second reads of outside CT for parathyroid adenoma operative planning.

**Figure 2:**
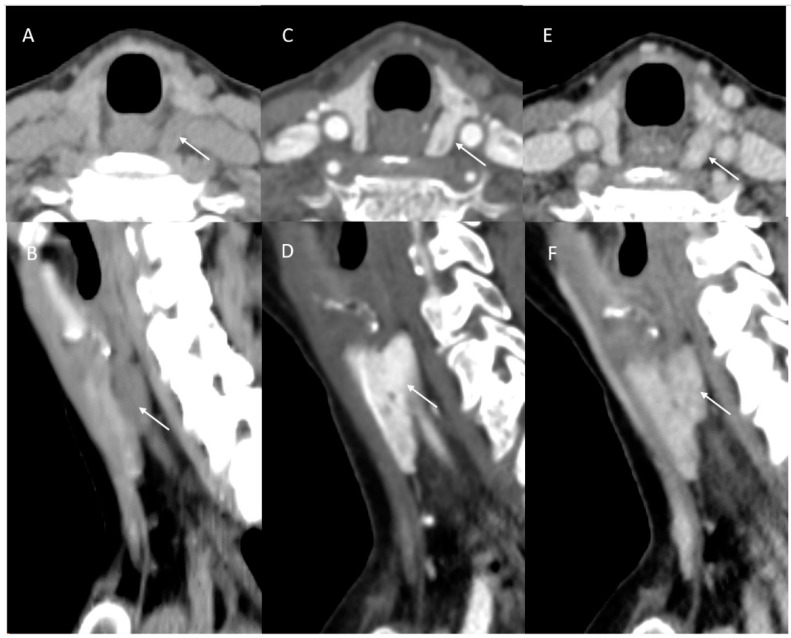
Parathyroid adenoma CT: initial outside and second read discrepancy. Outside neck CT was performed with the parathyroid protocol for the indication of primary hyperparathyroidism in a 73-year-old woman. The initial outside radiology impression was “Normal exam”. The second read identified the 19 mm lesion deep to the left thyroid lobe (arrows) which was hypoattenuating compared to the thyroid gland on non-contrast ((**A**), axial, (**B**), sagittal), avidly enhancing during arterial phase (**C**,**D**) and exhibiting slight earlier washout compared to the thyroid on delayed phase (**E**,**F**). This was confirmed intraoperatively with indocyanine green hyperfluorescence, resected and pathologically confirmed as hypercellular parathyroid adenoma on frozen section and final pathology.

The initial outside radiology report was correct for localization and multi-gland involvement in 44 of the 74 (59%) patients with operative and pathology results. The accuracy of outside neuroradiologist reports and outside diagnostic radiologist reports was similar (X^2^ = 0.0826, *p* = 0.78). Second reads of the same outside CT exams by neuroradiologists with high-volume experience with parathyroid adenoma imaging correctly identified candidate parathyroid adenomas laterality or bilaterality in an additional 18 of the 74 (24%) patients that were incorrectly or incompletely identified on the initial outside report (Figure 1). This included identifying adenoma(s) in 12 patients that did not have a candidate lesion identified on the initial outside read (Figure 2), multi-gland involvement in 3 patients for whom the initial outside report indicated solitary adenoma, single-gland involvement in 2 patients for whom the outside report indicated multi-gland involvement, and 1 patient with parathyroid adenoma contralateral to the initial reported location. Together, the total sensitivity for the initial and second read combined was 83%. 

### 3.4. Incorrect or Incomplete Localization 

**Figure 3:**
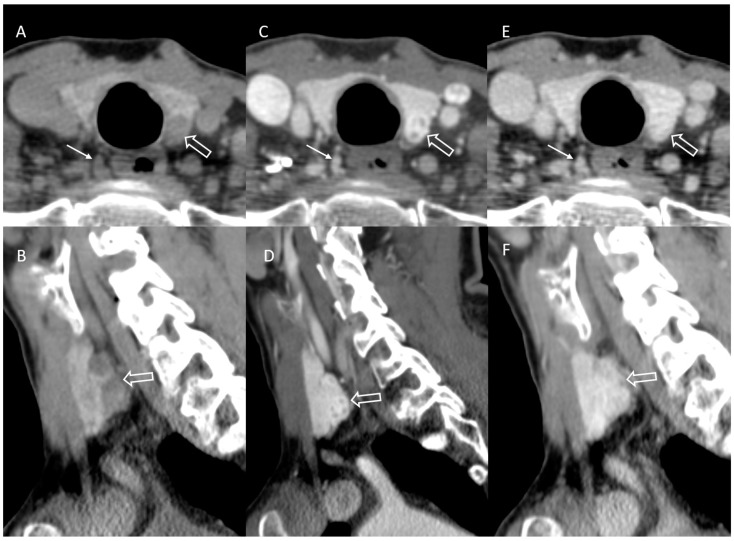
Incomplete initial outside and second read identification of parathyroid adenoma on CT. Outside neck CT was performed with the parathyroid protocol ((**A**,**B**) non-contrast, (**C**,**D**) arterial phase, (**E**,**F**) delayed phase) for the indication of primary hyperparathyroidism in a 67-year-old woman. Outside ultrasound and nuclear medicine sestamibi reports were negative, although the images were not available for comparison. The initial outside radiology impression and second read concurred and correctly identified a right paraesophageal parathyroid adenoma ((**A**,**C**,**E**) arrows). The initial report and second read both characterized the left thyroid lobe lesion as thyroid nodules ((**A**–**E**) open arrows). Bilateral neck exploration was performed, with frozen section and final pathology, confirming bilateral hypercellular parathyroid adenoma.

The initial outside read and second read were either incorrect or incompletely identified all lesions in multi-gland involvement for 12 patients. The majority included cases of “under calling”, identifying single lesions in fie patients with bilateral multi-gland involvement (Figure 3) and one patient with unilateral multi-gland involvement. For two patients, the initial outside read was negative. While a candidate parathyroid adenoma was identified on second read, neck exploration confirmed additional multi-gland parathyroid adenoma in these patients.

Both the initial outside and second read were incorrect in one patient for whom all removed candidate parathyroid glands were normal on pathology. There was one case where the initial read identified bilateral lesions, and a single parathyroid adenoma was confirmed; however, the location was discordant with the second read. Point-of-care ultrasound appropriately identified parathyroid adenoma preoperatively in two patients for whom the initial outside report and second read did not. 

Of the cases for which both the initial and second read were incomplete or resulted in incorrect localization, 36% were not performed with the multi-phase parathyroid protocol. 

### 3.5. Multi-Gland Involvement

In this cohort, 17 of the 74 (23%) patients had pathologically proven multi-gland involvement. The initial outside reports accurately identified multi-gland involvement in 4 patients, reflecting 8 of the 38 (21%) total lesions, and incorrectly reported 5 patients as having no parathyroid adenoma. Second reads correctly identified 26 of the 38 total lesions (68%) that were subsequently pathologically proven parathyroid adenoma. 

## 4. Discussion 

The majority of primary hyperparathyroidism cases are due to a single adenoma [11], and correct preoperative identification of involved parathyroid glands allows for targeted minimally invasive approaches. These consequently reduce surgical risks and operative time, minimize costs for patients and institutions, decrease postoperative pain and decrease hospital length of stay when compared to traditional four-gland parathyroidectomy and bilateral neck dissection [12]. 

In our series, the initial outside report was correct for localizing the laterality of parathyroid adenomas in 59% of the 74 patients who had surgical pathology results. This is lower than the sensitivity of >80% reported in larger case series [2,3,4]. However, this may be a more accurate measure of community-based radiology practice, rather than academic sub-specialty practices or high-volume parathyroid surgery centers that contribute to the literature. A limitation of this study is that other potential causes for discrepancy in accuracy cannot be assessed, as the initial workflow is external to our institution. Subspecialty neuroradiology training and certification was not a significant factor for accuracy for the outside radiologists. Additionally, imaging protocol and reconstructions alone did not account for the lower sensitivity, as second reads of the same outside CT scans correctly influenced surgical management in an additional 18 patients, raising the total sensitivity to 83%, on par with the literature.

Of the cases with incomplete or incorrect localization in this study both on the initial outside and second read, 36% were not performed with the multi-phase parathyroid protocol. Since parathyroid neck CT and routine neck CT share the same CPT codes, it is important that the practitioners specify on the orders that the CT scan be performed with the multi-phase parathyroid protocol neck CT. At our institution, we have implemented a unique order for parathyroid neck CT within the electronic medical record to specify this operative planning scan. Repeating imaging at high-volume centers after initially negative results can enhance localization sensitivity to as much as 92% [1,13], but incurs additional patient cost, contrast and radiation exposure. 

While approximately 15–20% of patients with primary hyperparathyroidism will have multi-gland involvement [14], accurate detection of multi-gland parathyroid adenoma continues to be a limitation of preoperative imaging across modalities, reported as 58–59% [3,4]. Multi-gland adenoma detection increased from 21% on the initial outside report to 68% with a second read of the same CT scans. It is unclear if this is related to a knowledge gap amongst community radiologists regarding multi-gland involvement or related to “satisfaction of search” [15] once a candidate parathyroid adenoma is identified. Re-review of the CT scans after pathology results were available confirmed that adenomas missed both on the initial outside and second read did not exhibit characteristic enhancement or contrast washout patterns when able to be identified retrospectively. 

There is a wide variety of policies across medical centers regarding if, when and how to provide second reads of outside imaging [16], the cost–benefit ratio and variations in double reading [17], and questioning if the second reads are actually desired or reviewed by the treatment team [18]. Parathyroid neck CT has a somewhat unique niche within this broader discussion, as it is truly an operative planning study and not indicated to make the diagnosis [8]. Therefore, it is not necessary to have the scan completed prior to initiating surgical referral, and imaging may best be performed within a radiology practice that performs a high volume of parathyroid neck CTs. However, insurance steering and plan networks may limit the location where patients can have imaging. In those cases, the ability for surgeons to have an internal review via a second-read process can be beneficial. 

A multidisciplinary effort is required for operative success, as accurately locating adenomas is essential for favorable surgical results. Thus, the importance of precise preoperative imaging cannot be understated.

## 5. Conclusions 

Endocrine surgeons should be aware that community-based radiology interpretation of neck CT scan may be less sensitive than reported series from academic and high-volume practices. In the present study, interpretation via second reads of outside CT scans by a neuroradiologist engaged with the endocrine surgery service line increased the sensitivity of detecting candidate lesions, both for single-gland and multi-gland involvement. While it is preferred to have preoperative imaging and interpretation within the same high-volume center as the endocrine surgeon for consistency of imaging quality, experience and communication, radiology second reads deserve financial and service line support when that is not possible given the impact on surgical planning and patient care. 

## Figures and Tables

**Table 1:** Demographic information of study population (*n* = 74).

	Number of Patients	Percentage of Patients
**Gender**		
Female	58	78%
Male	16	22%
**Age**		
25–45	4	5%
45–65	23	31%
65+	47	64%

## Data Availability

The original contributions presented in this study are included in the article. Further inquiries can be directed to the corresponding author(s).

## Abbreviations

The following abbreviations are used in this manuscript:

CT	Computed tomography
CPT	Current Procedure Terminology
PTH	Parathyroid hormone

## References

[1] Hunter G.J., Schellingerhout D., Vu T.H., Perrier N.D., Hamberg L.M. (2012). Accuracy of Four-dimensional CT for the Localization of Abnormal Parathyroid Glands in Patients with Primary Hyperparathyroidism. Radiology.

[2] Bahl M., Sepahdari A.R., Sosa J.A., Hoang J.K. (2015). Parathyroid Adenomas and Hyperplasia on Four-dimensional CT scans: Three Patterns of Enhancement Relative to the Thyroid Gland Justify a Three-Phase Protocol. Radiology.

[3] Yeh R., Tay Y.D., Tabacco G., Dercle L., Kuo J.H., Bandeira L., McManus C., Leung D.K., Lee J.A., Bilezikian J.P. (2019). Diagnostic Performance of 4D CT and Sestamibi SPECT/CT in Localizing Parathyroid Adenomas in Primary Hyperparathyroidism. Radiology.

[4] Beland M.D., Mayo-Smith W.W., Grand D.J., Machan J.T., Monchik J.M. (2011). Dynamic MDCT for localization of occult parathyroid adenomas in 26 patients with primary hyperparathyroidism. AJR Am. J. Roentgenol..

[5] Hinson A.M., Lee D.R., Hobbs B.A., Fitzgerald R.T., Bodenner D.L., Stack B.C. (2015). Preoperative 4D CT Localization of Nonlocalizing Parathyroid Adenomas by Ultrasound and SPECT-CT. Otolaryngol. Head Neck Surg..

[6] Bellamkonda N., Highland J., McCrary H.C., Slattery L., King B., Teames C., LeBaron K., Wiggins R.H., Abraham D., Hunt J.P. (2024). Four-Dimensional Computed Tomography for Parathyroid Adenoma Localization: A Pre-Operative Imaging Protocol. Ann. Otol. Rhinol. Laryngol..

[7] Hoang J.K., Williams K., Gaillard F., Dixon A., Sosa J.A. (2016). Parathyroid 4D-CT: Multi-institutional International Survey of Use and Trends. Otolaryngol. Head Neck Surg..

[8] Bahl M. (2019). Preoperative Parathyroid Imaging: Trends in Utilization and Comparative Accuracy of Sonography, Scintigraphy, and 4-Dimensional Computed Tomography. J. Comput. Assist. Tomogr..

[9] Khoshpouri P., Khoshpouri P., Yousem K.P., Yousem D.M. (2020). How do American radiology institutions deal with second opinion consultations on outside studies?. AJR Am. J. Roentgenol..

[10] Geijer H., Geijer M. (2018). Added value of double reading in diagnostic radiology, a systematic review. Insights Imaging.

[11] Heinz S.A., Kwee T.C., Yakar D. (2020). Unread Second-Opinion Radiology Reports: A Potential Waste of Health Care Resources. AJR Am. J. Roentgenol..

[12] Bilezikian J.P., Bandeira L., Khan A., Cusano N.E. (2018). Hyperparathyroidism. Lancet.

[13] Zander D., Bunch P.M., Policeni B., Juliano A.F., Carneiro-Pla D., Dubey P., Gule-Monroe M.K., Hagiwara M., Hoang J.K., Jain V. (2021). ACR Appropriateness Criteria^®^ Parathyroid Adenoma. J. Am. Coll. Radiol..

[14] Wilhelm S.M., Wang T.S., Ruan D.T., Lee J.A., Asa S.L., Duh Q.-Y., Doherty G.M., Herrera M.F., Pasieka J.L., Perrier N.D. (2016). The American Association of Endocrine Surgeons Guidelines for Definitive Management of Primary Hyperparathyroidism. JAMA Surg..

[15] Walker M., Silverberg S. (2018). Primary hyperparathyroidism. Nat. Rev. Endocrinol..

[16] Sosa J.A., Udelsman R. (2003). Minimally invasive parathyroidectomy. Surg. Oncol..

[17] Rodgers S.E., Hunter G.J., Hamberg L.M., Schellingerhout D., Doherty D.B., Ayers G.D., Shapiro S.E., Edeiken B.S., Truong M.T., Evans D.B. (2006). Improved preoperative planning for directed parathyroidectomy with 4-dimensional computed tomography. Surgery.

[18] Adamo S.H., Gereke B.J., Shomstein S., Schmidt J. (2021). From “satisfaction of search” to “subsequent search misses”: A review of multiple-target search errors across radiology and cognitive science. Cogn. Res..

